# Supplementary material for: Generation of Induced Pluripotent Stem Cells and Neuroepithelial Stem Cells from a Family with the Pathogenic Variant p.Q337X in Progranulin
Source: Int J Mol Sci. 2025 Nov 21;26(23):11242. doi: 10.3390/ijms262311242 (PMC12692286; doi:10.3390/ijms262311242)
Supplement: Supplementary file 1 [file ijms-26-11242-s001.zip › ijms-3935649-supplementary.pdf]

*Supplementary Material*

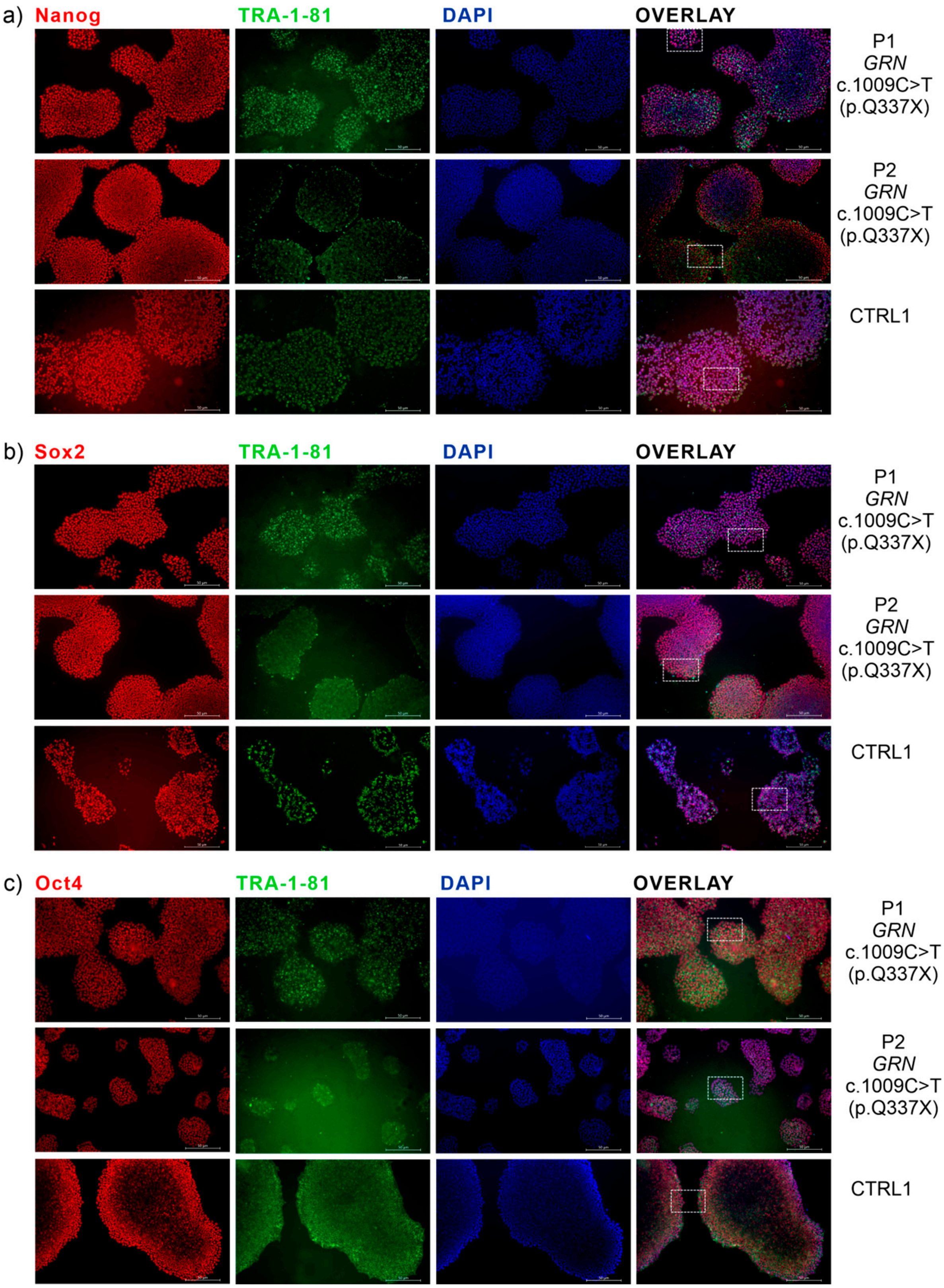


Figure S1. Immunostaining for pluripotency markers (original split and merged images); a) Nanog and TRA-1-81, b) Sox2 and TRA-1-81, c) Oct4 and TRA-1-81. The regions outlined with a white dashed line were used to generate the cropped areas shown in Fig. 1a of the manuscript. Nanog, Sox2, Oct4: secondary antibody Alexa Flour 555 red; TRA1-1-81: secondary antibody Alexa Flour 488 green, DAPI (blue). Scale bar: 50 µm

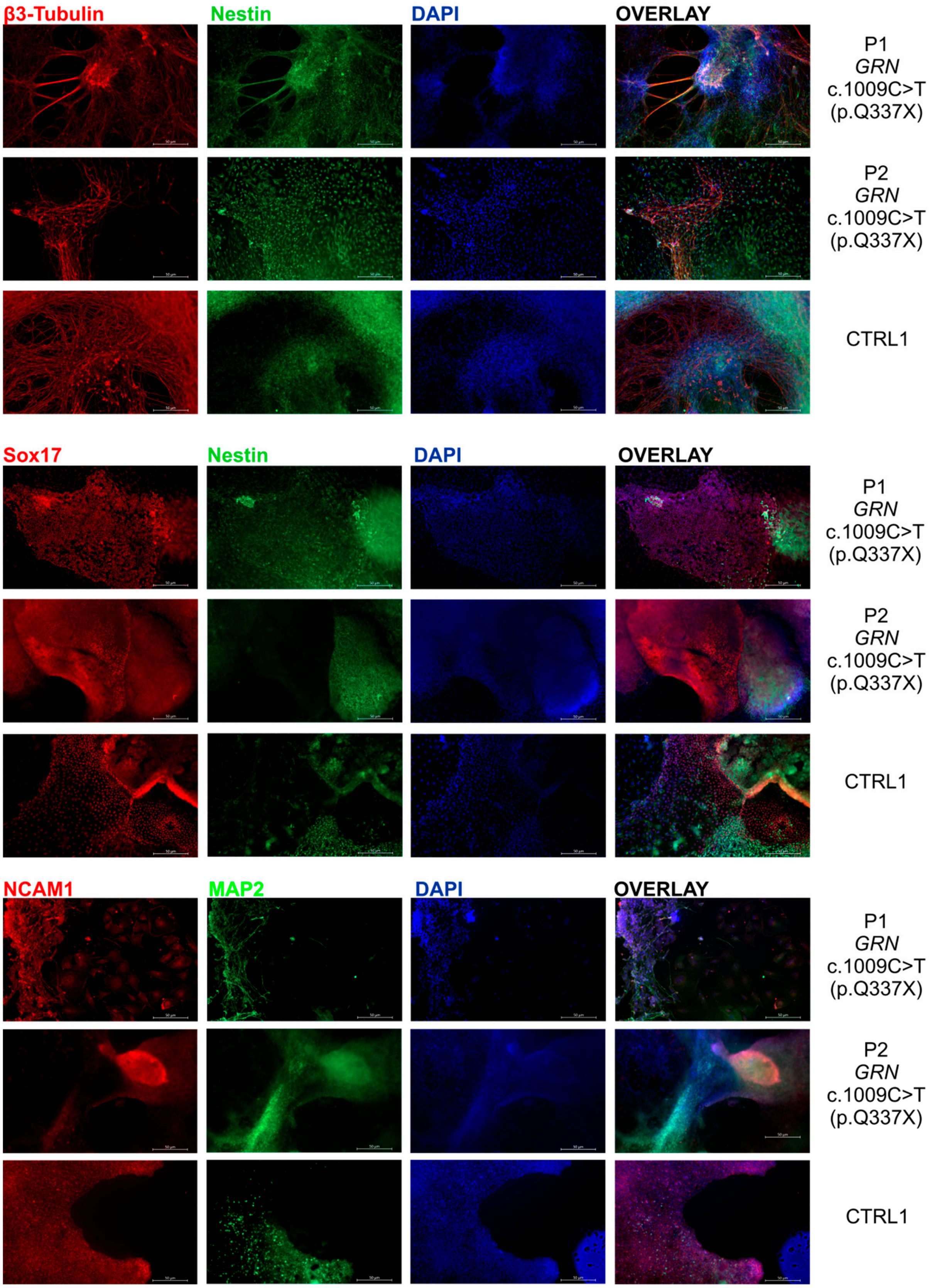


Figure S2. The split channels corresponding to merged images presented in Figure 2a. Immunostaining for β3-Tubulin, Sox17, NCAM1 (red, secondary antibody Alexa Flour 555 red), Nestin, MAP2 (green, secondary antibody Alexa Flour 488 green), DAPI (blue), and overlay images from these three channels. Scale bar: 50 μm;

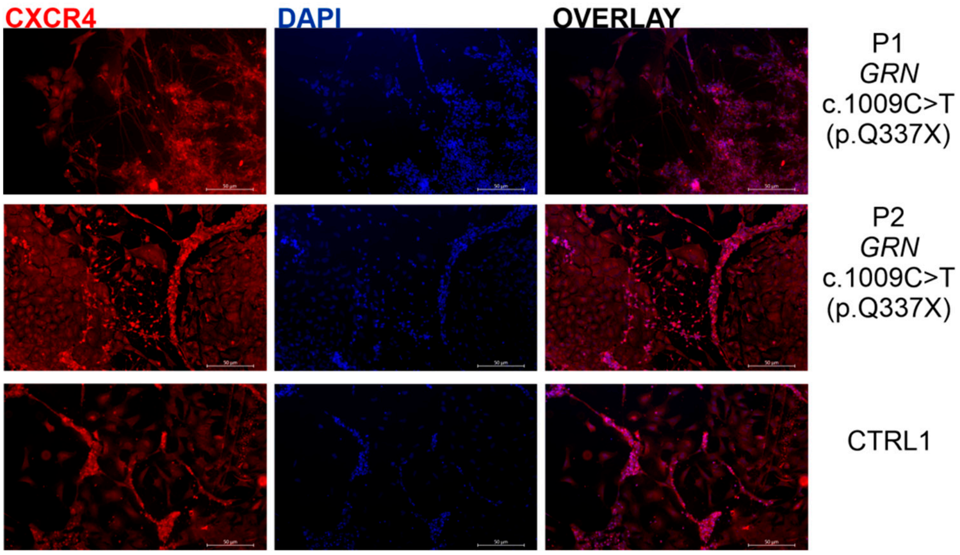


Figure S2.continued. Shows the corresponding split channels for overlay images presented in Figure 2a. Immunostaining for CXCR4 (red, secondary antibody Alexa Flour 555 red), and DAPI (blue) and overlay images from these two channels. Scale bar: 50 µm.

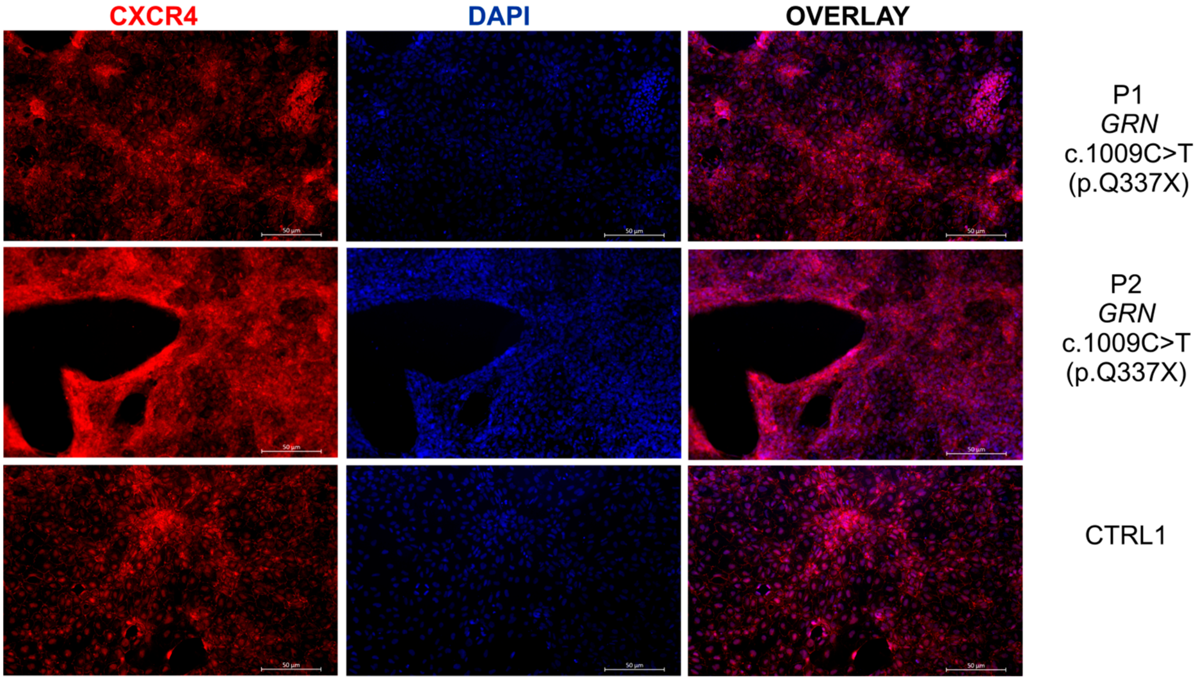


Figure S3. Immunostaining of mesodermal lineage cells for the mesodermal marker CXCR4 in P1, P2 and CTRL1 iPSC lines.

In parallel with embryoid body formation, P1, P2 and CTRL1 iPSC lines were differentiated into the three germ layers using a STEMdiff Trilineage Differentiation Kit (STEMCELL Technologies). In contrast to the spontaneous and highly heterogeneous formation of 3D embryoid bodies over more than 21 days, this method utilizes defined media to achieve directed differentiation into ectoderm, mesoderm, and endoderm within just 5–7 days. Mesodermal marker CXCR4 was visualized by immunostaining. CXCR4 is primarily a membrane-bound receptor, a G protein-coupled receptor (GPCR) that resides on the plasma membrane where it binds its ligand [12]. Cytoplasmic localization of CXCR4 is also observed due to constitutive endocytosis [12].

a)

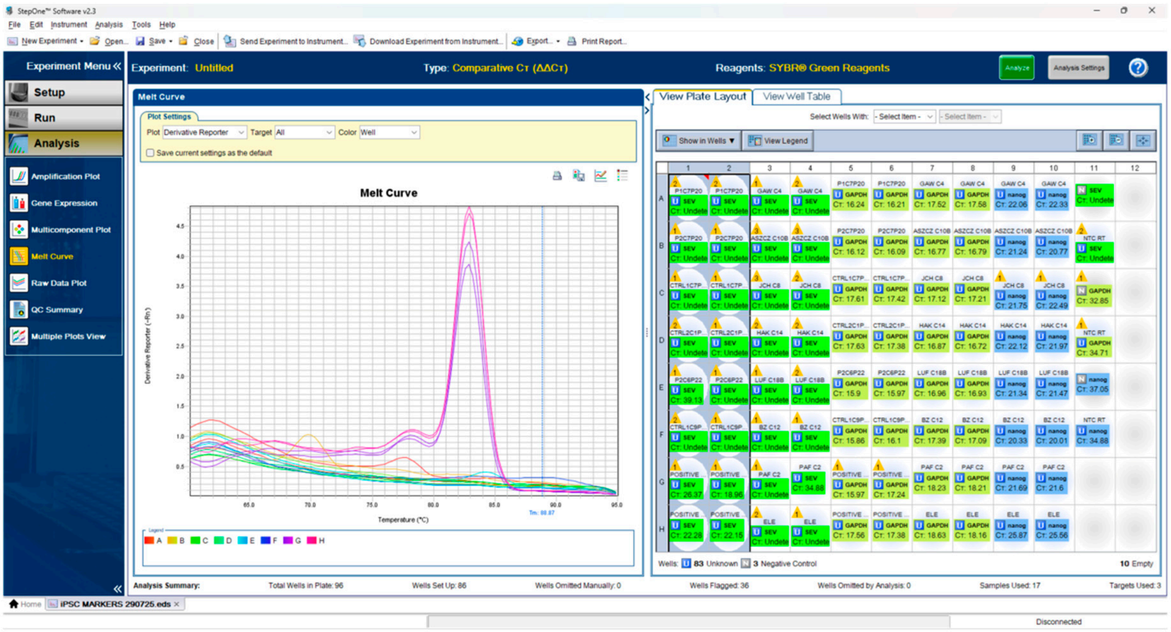


b)

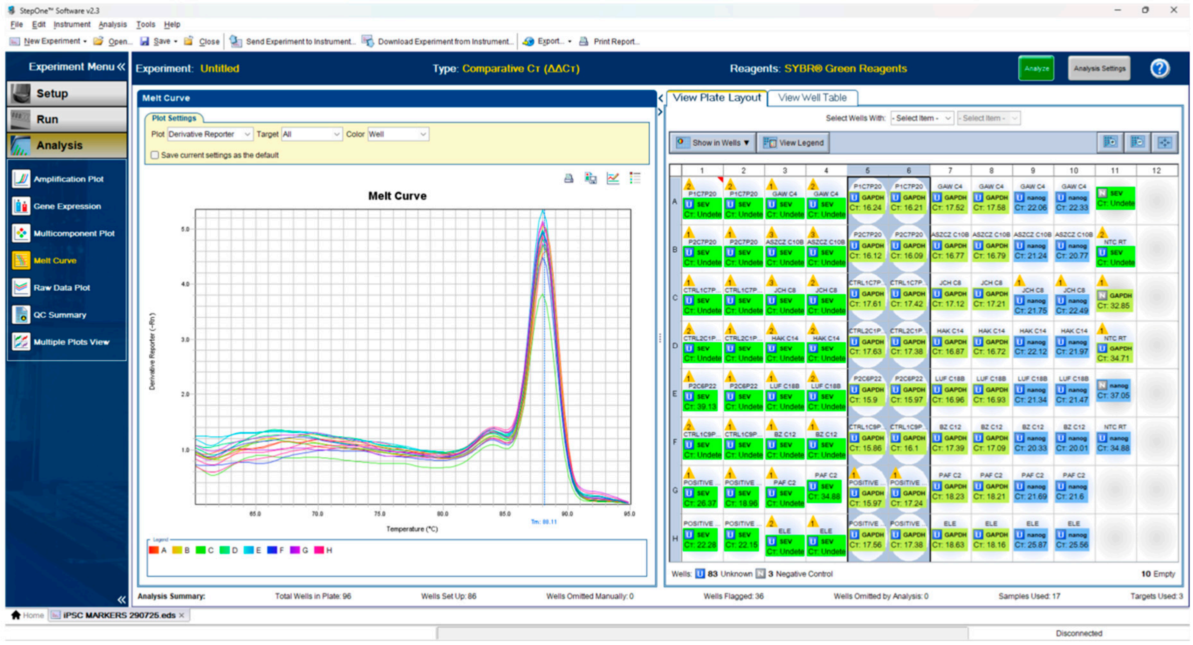


Figure S4. Real-time PCR analysis of Sendai virus expression. cDNA derived from various clones of P1, P2, CTRL1, and CTRL2 iPSC lines was analyzed by RT-PCR and revealed no residual expression of Sendai virus-derived reprogramming vectors. (a) The screenshot displays melt curve analysis of PCR products amplified using primers specific to the Sendai virus. No amplification was observed in wells A–F (1–2), corresponding to the iPSC clones P1, P2, CTRL1, and CTRL2, while early-passage iPSCs—still expressing viral sequences—served as positive controls (wells G–H, 1–2) showed distinct amplification peaks. (b) The second screenshot shows melt curve analysis using primers targeting the housekeeping gene GAPDH. All wells (A–H, 1–2) demonstrated clear amplification peaks, confirming the integrity of the cDNA and PCR reaction.

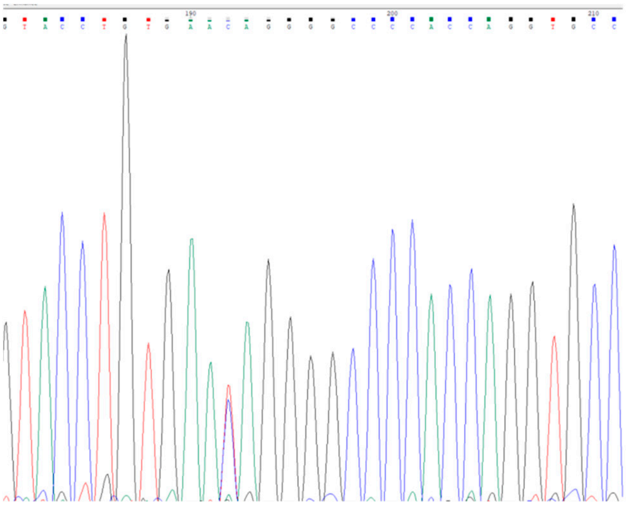

P1
*GRN* c.1009C>T

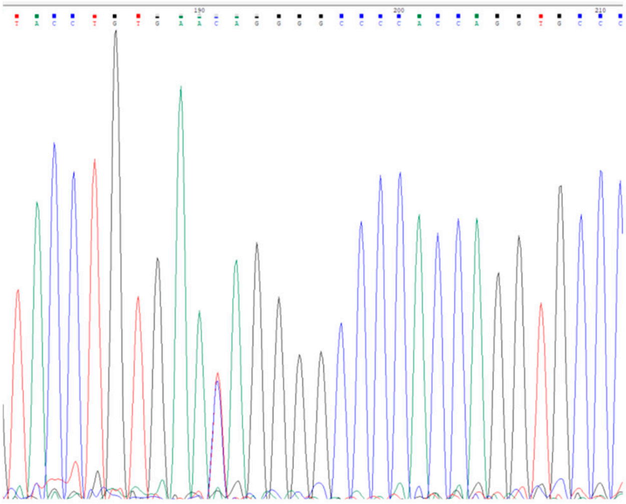

P2
*GRN* c.1009C>T

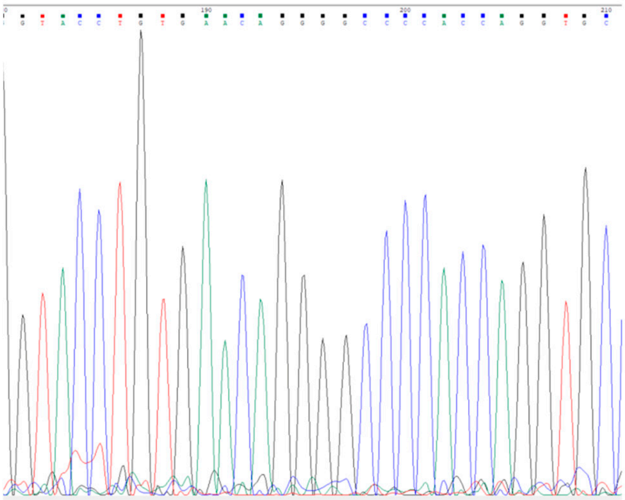

CTRL1

Figure S5. Sanger sequencing. Confirmation of the presence of the pathogenic variant c.1009C>T in iPSC lines derived from mutation carriers (P1 and P2) and wild-type sequence in the non-carrier (CTRL1).

Supplementary Material and Methods

Trilineage Differentiation

The potential of the established iPSC lines to differentiate into the three germ layers was in parallel assessed using STEMdiff Trilineage Differentiation Kit (STEMCELL Technologies) according to the manufacturer instructions.

Karyotype analysis - G-banding staining

iPSC cells (various clones of P1, P2, and CTRL1-4 iPSC lines) were cultured for 72-96 hours on 60 mm dishes coated with vitronectin in 3-5 ml of E8 medium until reaching 60–70% confluence. On the day of the experiment, E8 medium was changed in the morning to fresh one, and after 1–2 hours, colcemid was added (final concentration 0.1 µg/ml) and the cells were further incubated. After 1–1.5 hours, the cells were rinsed with 1.5 ml sterile PBS, then detached for approximately 4 minutes using a mixture of 700 µl Accutase + 700 µl EDTA, followed by the addition of double the volume of E8 medium. Then the cells were collected into 15 ml falcons and centrifuged for 5 minutes at 1000 rpm. After discarding the supernatant, the cell pellet was resuspended in 2 ml of warm 0.075M KCl (37°C) added dropwise using a Pasteur pipette. Tubes were gently flicked 2–3 times to help the KCl penetrate the cells. Tubes were incubated at 37°C for 25–30 minutes. After this time, 1 ml of fresh, ice-cold fixative (3:1 methanol:glacial acetic acid 99.5%) was added and cells were incubated at room temperature for 15 minutes. Tubes were centrifuged for 10 minutes at 1100 rpm. After each centrifugation with fixative, supernatant was removed the cell pellet was resuspended in 2- 4 ml of ice-cold fixative (3:1), and incubated at room temperature for 10 minutes. For final fixation, 5 ml of ice-cold fixative (2:1) was added and falcons were stored at 4°C until analysis. Karyotype analysis was performed on microscope slides prepared from the obtained fixed iPSC suspension. To visualize the characteristic GTG banding pattern (Giemsa Trypsin G-banding) in metaphase chromosomes, a standard protocol involving enzymatic digestion followed by differential staining was employed. The slides were briefly incubated in a 0.025% trypsin solution, then rinsed sequentially in PBS and Sørensen's buffer. Staining was carried out using a Giemsa dye solution adjusted to pH 6.8. Over twenty metaphases were analyzed for each sample. Cytogenetic analysis and karyotyping were performed with a light microscope (Nikon Eclipse 50i) integrated with LUCIA Cytogenetics Imaging Software.

*Generation of neuroepithelial stem cells (NES) and neuronal differentiation*

The cells were cultured at 37◦C in 5% CO2. Briefly, on Day 0, 200,000 iPSCs were seeded into each well of a vitronectin-coated 12-well plate in E8 medium supplemented with 10 µmol Y-27632 ROCK Inhibitor. On Day 1, the medium was replaced with pre-warmed neural induction medium-1, composed of DMEM/F-12 with GlutaMAX, 20% KnockOut Serum Replacement (KOSR), 1x non-essential amino acids, 100 µM 2-mercaptoethanol, and 100U/mL penicillin-streptomycin, 500ng/mL Noggin, 10µM SB431542, and 3.3 µM CHIR99021 [14,35]. The media was refreshed daily from up to Day 4. On Day 5, cells were split using TrypLE into 12-well plates coated with poly-L-ornithine (Sigma-Aldrich) and laminin 2020 (Sigma-Aldrich). From Day 5 onward, media was changed daily through Day 11 with gradual transition from KOSR-based to N2B27-based formulations according to the media composition in the published protocol [14,35]. On day 11 and 12 the medium contained 100% N2B27 medium: DMEM/F-12 with GlutaMAX, neurobasal, 2-mercaptoethanol, N2, B27, penicillin-streptomycin, and CHIR99021, while SB431542 and hNoggin was omitted [14,35]. On Day

12, cells were dissociated using TrypLE and transferred into poly-L-ornithine (100 ug /mL, Sigma-Aldrich #P36655) and laminin 2020 (1 ug/ml, Sigma-Aldrich, #L2020)- coated 12-well plates with NES growth medium (containing DMEM/F-12+GlutaMAX, N2, B27, 10 ng/mL bFGF (Thermo Fisher Scientific, Gibco), and 10ng/mL EGF (PreproTech), and penicillin-streptomycin [14]. NES cultures were passaged upon reaching 80-90% confluency and maintained with daily media changes. NES cells were passaged at a 1:2 ratio for five passages.

Assessment of iPSC Line Purity

To assess the purity of the obtained iPSC lines, fluorescence images of Nanog- and DAPI-stained nuclei were acquired using a Zeiss Axioexaminer.Z1 microscope. Several fields per line were captured under identical exposure conditions (P1- 10 images, P2- 10 images, CTRL1- 24 images).
Original RGB images were split into individual color channels, with DAPI-stained nuclei represented in the blue channel and Nanog-stained nuclei in the red channel. Nuclei were segmented from the blue channel using Cellpose (version 3.1.1.1) with the cyto3 model and automatic calibration.
Background signal in the red channel was separated from the masks of segmented nuclei generated by Cellpose using ImageJ (version 1.54p).

In images where local culture density was too high for automated processing, those regions were manually excluded using thresholding to prevent erroneous data inclusion.

Background intensity values were collected, and sigma clipping with a threshold of 1.5σ was applied to exclude residual debris. The maximum remaining intensity value was subtracted from all pixel values within each nucleus to reduce overall intensity. Nuclei with a maximum normalized intensity greater than zero were classified as positive, while those with zero intensity were classified as negative. Additionally, a Z-score was computed for each nucleus to identify potential anomalies, and nuclei flagged as anomalous were excluded from the count of positive reactions.

Table S1. Primers used in the study

| Target sequence | Primer sequence | reference |
|---|---|---|
| *GRN* (mRNA) | TTGCTGCTGCCCAAGGAC<br><br>GCCATTTGTCCAGAAGGGGA | [34] |
| *GAPDH* | GTTCGACAGTCAGCCGCATC<br><br>GGAATTTGCCATGGGTGGA | [36] |
| Sendai Virus | GGATCACTAGGTGATATCGAGC<br>ACCAGACAAGAGTTTAAGAGATATGTATC | [15] |
| iPSC markers | | |
| *NANOG* | CATGAGTGTGGATCCAGCTTG<br>CCTGAATAAGCAGATCCATGG | [15] |
| *OCT4* | CTCACCCTGGGGGTTCTATT<br>CTCCAGGTTGCCTCTCACTC | [15] |
| germ layer markers | | |
| *TBXT (mesoderm)* | AACGGCAGGAGGATGTTTC<br>GTTCACGTACTTCCAGCGGT | [37] |
| *FOXA2 (endoderm)* | GGGAGCGGTGAAGATGGAAG | [37] |

| | | |
|---|---|---|
| | TCATGCCGTTCATCCCCAG | |
| NES markers | | |
| *SOX1* | TGGATGAAGGACAAAGACCA<br>TTTGGTTCAGCGATTGTGTT | [15] |
| *PLAGL1* | AACGAGACTGGGACTATGGC<br>CACGCGTTCATTAAATTTGG | |
| *MKI67* | TGGTAATGCACACTCCACCT<br>TTGTGCCTTCACTTCCACAT | |

Table S2. Antibodies used in the study

| Antibody/host | dilution | host | Catalog number | source |
|---|---|---|---|---|
| Pluripotency markers | | | | |
| anti- Oct-4A (C30A3) | 1:400 | rabbit | #2840 | Cell Signaling Technology, Danvers, MA,USA |
| anti-Sox2 (D6D9) XP® | 1:400 | rabbit | #3579 | Cell Signaling Technology, Danvers, MA,USA |
| anti-Nanog (D73G4) XP® | 1:400 | rabbit | #4903 | Cell Signaling Technology, Danvers, MA,USA |
| anti- TRA-1-81 | 1:400 | mouse | #4745 | Cell Signaling Technology, Danvers, MA,USA |
| Ectoderm markers | | | | |
| anti-β3-Tubulin (D71G9) XP® | 1:400 | rabbit | #74597 | Cell Signaling Technology, Danvers, MA,USA |
| anti-Nestin clone 10C2 | 1:1000 | mouse | MAB5326 | Sigma-Aldrich |
| anti-MAP2 | 1:400 | mouse | M4403 | Merck |
| Mesoderm markers | | | | |
| anti-CXCR4 | 1:200 | rabbit | DF8046 | Affinity Biosciences |
| anti-NCAM1 | 1:300 | rabbit | DF7832 | Affinity Biosciences |
| Endoderm markers | | | | |
| Sox17 | 1:1000 | rabbit | DF9090 | Affinity Biosciences |
| Secondary antibodies | | | | |
| Goat anti-Rabbit IgG (H+L) Cross-Adsorbed Secondary Antibody, Alexa Fluor™ 555 | 1:1000 | rabbit | A21428 | Invitrogen |
| Goat anti-Mouse IgG (H+L) Cross-Adsorbed Secondary Antibody, Alexa Fluor™ 488 | 1:1000 | mouse | A11001 | Invitrogen |
